# Supplementary material for: Score Predictor Factor Analysis: Reproducing Observed Covariances by Means of Factor Score Predictors
Source: Front Psychol. 2019 Aug 16;10:1895. doi: 10.3389/fpsyg.2019.01895 (PMC6707334; doi:10.3389/fpsyg.2019.01895)
Supplement: Supplementary file 1 [file Table_1.docx]

Supplementary Material

**Score predictor factor analysis:**

**Reproducing observed covariances by means of factor score predictors**

André Beauducel^1*^, Norbert Hilger^1^

* Correspondence: André Beauducel: beauducel@uni-bonn.de

# R-Code for unrotated PCA, MFA and SPFA

# This R-script needs the "psych" package provided by William Revelle at

# https://personality-project.org/r/psych

# The correlation matrix of the 15-Items is

Sig = matrix(c(

1.000, 0.000, 0.000, 0.000, 0.000, 0.000, 0.000, 0.000, 0.000, 0.000, 0.000, 0.000, 0.000, 0.000, 0.000,

0.250, 1.000, 0.000, 0.000, 0.000, 0.000, 0.000, 0.000, 0.000, 0.000, 0.000, 0.000, 0.000, 0.000, 0.000,

0.250, 0.250, 1.000, 0.000, 0.000, 0.000, 0.000, 0.000, 0.000, 0.000, 0.000, 0.000, 0.000, 0.000, 0.000,

0.250, 0.250, 0.250, 1.000, 0.000, 0.000, 0.000, 0.000, 0.000, 0.000, 0.000, 0.000, 0.000, 0.000, 0.000,

0.250, 0.250, 0.250, 0.300, 1.000, 0.000, 0.000, 0.000, 0.000, 0.000, 0.000, 0.000, 0.000, 0.000, 0.000,

0.000, 0.000, 0.000, 0.000, 0.000, 1.000, 0.000, 0.000, 0.000, 0.000, 0.000, 0.000, 0.000, 0.000, 0.000,

0.000, 0.000, 0.000, 0.000, 0.000, 0.250, 1.000, 0.000, 0.000, 0.000, 0.000, 0.000, 0.000, 0.000, 0.000,

0.000, 0.000, 0.000, 0.000, 0.000, 0.250, 0.250, 1.000, 0.000, 0.000, 0.000, 0.000, 0.000, 0.000, 0.000,

0.000, 0.000, 0.000, 0.000, 0.000, 0.250, 0.250, 0.250, 1.000, 0.000, 0.000, 0.000, 0.000, 0.000, 0.000,

0.000, 0.000, 0.000, 0.000, 0.000, 0.250, 0.250, 0.250, 0.300, 1.000, 0.000, 0.000, 0.000, 0.000, 0.000,

0.000, 0.000, 0.000, 0.000, 0.000, 0.000, 0.000, 0.000, 0.000, 0.000, 1.000, 0.000, 0.000, 0.000, 0.000,

0.000, 0.000, 0.000, 0.000, 0.000, 0.000, 0.000, 0.000, 0.000, 0.000, 0.250, 1.000, 0.000, 0.000, 0.000,

0.000, 0.000, 0.000, 0.000, 0.000, 0.000, 0.000, 0.000, 0.000, 0.000, 0.250, 0.250, 1.000, 0.000, 0.000,

0.000, 0.000, 0.000, 0.000, 0.000, 0.000, 0.000, 0.000, 0.000, 0.000, 0.250, 0.250, 0.250, 1.000, 0.000,

0.000, 0.000, 0.000, 0.000, 0.000, 0.000, 0.000, 0.000, 0.000, 0.000, 0.250, 0.250, 0.250, 0.300, 1.000

),nrow=15,ncol=15)

Sig <- Sig + t(Sig)

diag(Sig) <- 1

# PCA:

help <- eigen(Sig)

PC_eig <- (help$values)

Vec <- help$vectors

PC <- Vec%*%(diag(PC_eig)^0.5)

A <- PC[,1:3]

# Minres-FA (MFA):

Lm <- A

#compute H2=Mdiag(diag( make(nrow(L),nrow(L),1) )).

H2 <- diag( A%*%t(A) )

dif <- 1

funct <- 1

funct_o <- 1000

i <- 0

while (dif>0.000000001 & i<10000) {

i <- i+1

Srep <- Sig - diag(1, nrow(Sig),ncol(Sig)) + diag(H2)

help <- eigen(Srep)

K <- help$vectors[,1:ncol(A)]

Gam_eig <- diag(help$values[1:ncol(A)])

Lm <- K%*%(Gam_eig^0.5)

H2 <- diag(Lm%*%t(Lm))

dif <- sum(( Sig-Lm%*%t(Lm) - diag(Sig-Lm%*%t(Lm)) )^2) /(nrow(Sig)*(nrow(Sig)-1))

}

# Score-Predictor-FA (SPFA):

inv <- function(x) return(solve(x))

H2 <- diag(0, nrow(A),nrow(A) )

dif <- 1

i <- 0

while (dif>0.000000001 & i<10000) {

i <- i+1

Srep <- Sig - diag(1, nrow(Sig),ncol(Sig)) + diag(H2)

help <- eigen(Srep)

K <- help$vectors[,1:ncol(A)]

help2 <- eigen(t(K)%*%inv(Sig)%*%K)

# symmetric square root:

help2_12 <- help2$vectors %*% (diag(help2$values))^0.5 %*% t(help2$vectors)

# Equation 12, symmetric square root:

Ls <- K%*%inv(help2_12)

H2 <- diag(Ls%*%t(Ls))

dif <- sum(( Sig-Ls%*%t(Ls) - diag(diag(Sig-Ls%*%t(Ls))) )^2) /(nrow(Sig)*(nrow(Sig)-1))

}

# Equation 21:

Rho_A <- t(Lm)%*%A%*%inv(t(A)%*%A)

Rho_Lm <- diag(diag(t(Lm)%*%inv(Sig)%*%Lm)^0.5)

# Equation 22:

Rho_Ls <- t(Lm)%*%inv(Sig)%*%Ls %*%inv(diag(diag(t(Ls)%*%inv(Sig)%*%Ls)))^0.5

# Rho PCA:

Rho_A

# Rho MFA:

Rho_Lm

# Rho SPFA:

Rho_Ls

"PCA:"

print(A, digits=3)

"MFA:"

print(Lm, digits=3)

"SPFA:"

print(Ls, digits=3)

# R-Code for Varimax-rotated PCA, MFA and SPFA based on the correlations of the empirical example

# This R-script needs the "psych" package provided by William Revelle at

# https://personality-project.org/r/psych

# and the "GPArotation" package provided by Coen Bernaards and Robert Jennrich at

# https://cran.r-project.org/web/packages/GPArotation/index.html

install.packages("GPArotation")

library("GPArotation")

library(foreign)

# Empirical correlation matrix:

Sig = matrix(c(

1.000, 0.254, 0.227, 0.161, 0.280, 0.226, 0.171, 0.167, 0.109, 0.116, 0.201, 0.102,

0.254, 1.000, 0.239, 0.164, 0.229, 0.352, 0.074, 0.167, 0.103, 0.057, 0.152, 0.114,

0.227, 0.239, 1.000, 0.239, 0.246, 0.322, 0.046, 0.063, 0.087, 0.094, 0.196, 0.060,

0.161, 0.164, 0.239, 1.000, 0.208, 0.305, 0.002, 0.086, 0.180, 0.009, 0.149, 0.045,

0.280, 0.229, 0.246, 0.208, 1.000, 0.253, 0.157, 0.177, 0.092, 0.146, 0.125, 0.061,

0.226, 0.352, 0.322, 0.305, 0.253, 1.000,-0.007, 0.103, 0.165, 0.162, 0.161, 0.093,

0.171, 0.074, 0.046, 0.002, 0.157,-0.007, 1.000, 0.179, 0.243, 0.152, 0.220, 0.269,

0.167, 0.167, 0.063, 0.086, 0.177, 0.103, 0.179, 1.000, 0.117, 0.133, 0.110, 0.069,

0.109, 0.103, 0.087, 0.180, 0.092, 0.165, 0.243, 0.117, 1.000, 0.123, 0.283, 0.203,

0.116, 0.057, 0.094, 0.009, 0.146, 0.162, 0.152, 0.133, 0.123, 1.000, 0.129, 0.091,

0.201, 0.152, 0.196, 0.149, 0.125, 0.161, 0.220, 0.110, 0.283, 0.129, 1.000, 0.234,

0.102, 0.114, 0.060, 0.045, 0.061, 0.093, 0.269, 0.069, 0.203, 0.091, 0.234, 1.000

),nrow=12,ncol=12)

# PCA:

help <- eigen(Sig)

PC_eig <- (help$values)

Vec <- help$vectors

PC <- Vec%*%(diag(PC_eig)^0.5)

#### WITH KAISER-NORMALIZATION

#### SELECTION OF SOLUTION WITH GREATEST VARIMAX FROM UNROTATED LOADINGS AND 10 RANDOM #### START LOADINGS

#### additions to code for GPArotation (Bernaards & Jennrich, 2005):

#### Kaiser-normalization of loadings before rotation

#### loops for selecting best solution from identity-T and 10 random Ts

### settings:

n_fac <- 2

n_var <- 12

n_random <-10

n_iter <- 250

### get unrotated loading matrix A:

A <- PC[,1:2]

### Kaiser-normalization: divide loadings by square root of communalities

A_sq <-A^2

H_sq <- rowSums(A_sq, na.rm = FALSE, dims = 1)

H_root <- H_sq^0.5

H_diag <- diag(H_root)

A_kais <- solve(H_diag)%*%A

A <- A_kais

### GPR-Varimax

### first use identity-T (unrotated start loadings)

GPR_ident <- GPForth(A, Tmat=diag(ncol(A)), normalize=FALSE, eps=1e-5, maxit=n_iter,

method="varimax", methodArgs=NULL)

A_sol1 <- GPR_ident[["loadings"]]

### reverse Kaiser-normalization after rotation:

A_sol1 <- diag(H_root)%*%A_sol1

### loop across 10 random start loadings and replace solution if Varimax criterion gets larger

### set preliminary varicrit and A solution to solution from T=identity:

A_sol <- A_sol1

vari_crit <- vari_1

for (i_random in 1:n_random){

Random.Start <- function(n_fac){

qr.Q(qr(matrix(rnorm(n_fac*n_fac),n_fac)))

}

T_rnd <- Random.Start(n_fac)

GPR_rand <- GPForth(A, Tmat=T_rnd, normalize=FALSE, eps=1e-5, maxit=n_iter,

method="varimax", methodArgs=NULL)

A_solr <- GPR_rand[["loadings"]]

### reverse Kaiser-normalization after rotation:

A_solr <- diag(H_root)%*%A_solr

### Varimax criterion for each solution from random start loadings:

A_sq <- A_solr^2

mn_A_sq1 <- colSums(A_sq)/n_var

mn_A_sq2 <- matrix(rep(mn_A_sq1,each=n_var), nrow=n_var, byrow=FALSE)

A_diff <- A_sq - mn_A_sq2

A_diff_sq <- A_diff^2

A_diff_cssq <- colSums(A_diff_sq, na.rm = FALSE, dims = 1)

var_A_sq <- A_diff_cssq/n_var

var_A_rssq <- sum(var_A_sq)

vari_rnd <- var_A_rssq/n_fac

### select solution with largest Varimax criterion:

if(vari_rnd > vari_crit){

vari_crit <- vari_rnd

A_sol <- A_solr

}

}

A_PCA <- A_sol

# Minres-FA (MFA):

Lm <- A

#compute H2=Mdiag(diag( make(nrow(L),nrow(L),1) )).

H2 <- diag( A%*%t(A) )

dif <- 1

funct <- 1

funct_o <- 1000

i <- 0

while (dif>0.000000001 & i<10000) {

i <- i+1

Srep <- Sig - diag(1, nrow(Sig),ncol(Sig)) + diag(H2)

help <- eigen(Srep)

K <- help$vectors[,1:ncol(A)]

Gam_eig <- diag(help$values[1:ncol(A)])

Lm <- K%*%(Gam_eig^0.5)

H2 <- diag(Lm%*%t(Lm))

dif <- sum(( Sig-Lm%*%t(Lm) - diag(Sig-Lm%*%t(Lm)) )^2) /(nrow(Sig)*(nrow(Sig)-1))

}

### get unrotated loading matrix A:

A <- Lm

### Kaiser-normalization: divide loadings by square root of communalities

A_sq <-A^2

H_sq <- rowSums(A_sq, na.rm = FALSE, dims = 1)

H_root <- H_sq^0.5

H_diag <- diag(H_root)

A_kais <- solve(H_diag)%*%A

A <- A_kais

### GPR-Varimax

### first use identity-T (unrotated start loadings)

GPR_ident <- GPForth(A, Tmat=diag(ncol(A)), normalize=FALSE, eps=1e-5, maxit=n_iter,

method="varimax", methodArgs=NULL)

A_sol1 <- GPR_ident[["loadings"]]

### reverse Kaiser-normalization after rotation:

A_sol1 <- diag(H_root)%*%A_sol1

### loop across 10 random start loadings and replace solution if Varimax criterion gets larger

### set preliminary varicrit and A solution to solution from T=identity:

A_sol <- A_sol1

vari_crit <- vari_1

for (i_random in 1:n_random){

Random.Start <- function(n_fac){

qr.Q(qr(matrix(rnorm(n_fac*n_fac),n_fac)))

}

T_rnd <- Random.Start(n_fac)

GPR_rand <- GPForth(A, Tmat=T_rnd, normalize=FALSE, eps=1e-5, maxit=n_iter,

method="varimax", methodArgs=NULL)

A_solr <- GPR_rand[["loadings"]]

### reverse Kaiser-normalization after rotation:

A_solr <- diag(H_root)%*%A_solr

### Varimax criterion for each solution from random start loadings:

A_sq <- A_solr^2

mn_A_sq1 <- colSums(A_sq)/n_var

mn_A_sq2 <- matrix(rep(mn_A_sq1,each=n_var), nrow=n_var, byrow=FALSE)

A_diff <- A_sq - mn_A_sq2

A_diff_sq <- A_diff^2

A_diff_cssq <- colSums(A_diff_sq, na.rm = FALSE, dims = 1)

var_A_sq <- A_diff_cssq/n_var

var_A_rssq <- sum(var_A_sq)

vari_rnd <- var_A_rssq/n_fac

### select solution with largest Varimax criterion:

if(vari_rnd > vari_crit){

vari_crit <- vari_rnd

A_sol <- A_solr

}

}

Lm <- A_sol

# Score-Predictor-FA (SPFA):

inv <- function(x) return(solve(x))

H2 <- diag(0, nrow(A),nrow(A) )

dif <- 1

i <- 0

while (dif>0.000000001 & i<10000) {

i <- i+1

Srep <- Sig - diag(1, nrow(Sig),ncol(Sig)) + diag(H2)

help <- eigen(Srep)

K <- help$vectors[,1:ncol(A)]

help2 <- eigen(t(K)%*%inv(Sig)%*%K)

# symmetric square root:

help2_12 <- help2$vectors %*% (diag(help2$values))^0.5 %*% t(help2$vectors)

# Equation 10, symmetric square root:

Ls <- K%*%inv(help2_12)

H2 <- diag(Ls%*%t(Ls))

dif <- sum(( Sig-Ls%*%t(Ls) - diag(diag(Sig-Ls%*%t(Ls))) )^2) /(nrow(Sig)*(nrow(Sig)-1))

}

### get unrotated loading matrix A:

A <- Ls

### Kaiser-normalization: divide loadings by square root of communalities

A_sq <-A^2

H_sq <- rowSums(A_sq, na.rm = FALSE, dims = 1)

H_root <- H_sq^0.5

H_diag <- diag(H_root)

A_kais <- solve(H_diag)%*%A

A <- A_kais

### GPR-Varimax

### first use identity-T (unrotated start loadings)

GPR_ident <- GPForth(A, Tmat=diag(ncol(A)), normalize=FALSE, eps=1e-5, maxit=n_iter,

method="varimax", methodArgs=NULL)

A_sol1 <- GPR_ident[["loadings"]]

### reverse Kaiser-normalization after rotation:

A_sol1 <- diag(H_root)%*%A_sol1

### loop across 10 random start loadings and replace solution if Varimax criterion gets larger

### set preliminary varicrit and A solution to solution from T=identity:

A_sol <- A_sol1

vari_crit <- vari_1

for (i_random in 1:n_random){

Random.Start <- function(n_fac){

qr.Q(qr(matrix(rnorm(n_fac*n_fac),n_fac)))

}

T_rnd <- Random.Start(n_fac)

GPR_rand <- GPForth(A, Tmat=T_rnd, normalize=FALSE, eps=1e-5, maxit=n_iter,

method="varimax", methodArgs=NULL)

A_solr <- GPR_rand[["loadings"]]

### reverse Kaiser-normalization after rotation:

A_solr <- diag(H_root)%*%A_solr

### Varimax criterion for each solution from random start loadings:

A_sq <- A_solr^2

mn_A_sq1 <- colSums(A_sq)/n_var

mn_A_sq2 <- matrix(rep(mn_A_sq1,each=n_var), nrow=n_var, byrow=FALSE)

A_diff <- A_sq - mn_A_sq2

A_diff_sq <- A_diff^2

A_diff_cssq <- colSums(A_diff_sq, na.rm = FALSE, dims = 1)

var_A_sq <- A_diff_cssq/n_var

var_A_rssq <- sum(var_A_sq)

vari_rnd <- var_A_rssq/n_fac

### select solution with largest Varimax criterion:

if(vari_rnd > vari_crit){

vari_crit <- vari_rnd

A_sol <- A_solr

}

}

Ls <- A_sol

A <-A_PCA

# Equation 10:

SRMRnd_PCA <- sum(( Sig- A%*%inv(t(A)%*%inv(Sig)%*%A)%*%t(A) - diag(diag(Sig- A%*%inv(t(A)%*%inv(Sig)%*%A)%*%t(A) )) )^2) /(nrow(Sig)*(nrow(Sig)-1))

SRMRnd_MFA <- sum(( Sig- Lm%*%inv(t(Lm)%*%inv(Sig)%*%Lm)%*%t(Lm) - diag(diag(Sig- Lm%*%inv(t(Lm)%*%inv(Sig)%*%Lm)%*%t(Lm) )) )^2) /(nrow(Sig)*(nrow(Sig)-1))

SRMRnd_SPFA <- sum(( Sig- Ls%*%inv(t(Ls)%*%inv(Sig)%*%Ls)%*%t(Ls) - diag(diag(Sig- Ls%*%inv(t(Ls)%*%inv(Sig)%*%Ls)%*%t(Ls) )) )^2) /(nrow(Sig)*(nrow(Sig)-1))

print(round(SRMRnd_PCA, digits = 4), digits=4)

print(round(SRMRnd_MFA, digits = 4), digits=4)

print(round(SRMRnd_SPFA, digits = 4), digits=4)

"PCA:"

print(round(A, digits = 2), digits=2)

"MFA:"

print(round(Lm, digits = 2), digits=2)

"SPFA:"

print(round(Ls, digits = 2), digits=2)
